# Impact of cumulative body mass index and cardiometabolic diseases on survival among patients with colorectal and breast cancer: a multi-centre cohort study

**DOI:** 10.1186/s12885-022-09589-y

**Published:** 2022-05-14

**Authors:** Mirjam Kohls, Heinz Freisling, Hadrien Charvat, Isabelle Soerjomataram, Vivian Viallon, Veronica Davila-Batista, Rudolf Kaaks, Renée Turzanski-Fortner, Krasimira Aleksandrova, Matthias B. Schulze, Christina C. Dahm, Helene Tilma Vistisen, Agnetha Linn Rostgaard-Hansen, Anne Tjønneland, Catalina Bonet, Maria-Jose Sánchez, Sandra Colorado-Yohar, Giovanna Masala, Domenico Palli, Vittorio Krogh, Fulvio Ricceri, Olov Rolandsson, Sai San Moon Lu, Konstantinos K. Tsilidis, Elisabete Weiderpass, Marc J. Gunter, Pietro Ferrari, Ursula Berger, Melina Arnold

**Affiliations:** 1 Institute for Medical Information Processing, Biometry and Epidemiology – IBE, LMU Munich, Munich, Germany; 2 Pettenkofer School of Public Health, Munich, Germany; 3 Cancer Surveillance Branch, International Agency for Research on Cancer – IARC/WHO, 150 cours Albert Thomas, 69372 CEDEX 08 Lyon, France; 4 Nutrition and Metabolism Branch, International Agency for Research on Cancer – IARC/WHO, 150 cours Albert Thomas, 69372 CEDEX 08 Lyon, France; 5 Division of Cancer Epidemiology, German Cancer Research Center (DKFZ), Heidelberg, Germany; 6 Nutrition, Immunity and Metabolism Senior Scientist Group, Department of Nutrition and Gerontology, German Institute of Human Nutrition Potsdam-Rehbruecke (DIfE), Nuthetal, Germany; 7 Institute of Nutritional Science, University of Potsdam, Potsdam, Germany; 8 Department of Molecular Epidemiology, German Institute of Human Nutrition Potsdam-Rehbruecke, Nuthetal, Germany; 9 Department of Public Health, Aarhus University, Aarhus, Denmark; 10 Danish Cancer Society Research Centre, Diet, Genes and Environment, Copenhagen, Denmark; 11 Department of Public Health, University of Copenhagen, Copenhagen, Denmark; 12 Unit of Nutrition, Environment and Cancer, Cancer Epidemiology Research Program, Institut Català d’Oncologia, Barcelona, Spain; 13 Escuela Andaluza de Salud Pública (EASP), Granada, Spain; 14 Instituto de Investigación Biosanitaria Ibs.GRANADA, Granada, Spain; 15 CIBER in Epidemiology and Public Health (CIBERESP), Madrid, Spain; 16 Universidad de Granada, Granada, Spain; 17 Department of Epidemiology, Murcia Regional Health Council, IMIB-Arrixaca, Murcia, Spain; 18 CIBER Epidemiología Y Salud Pública (CIBERESP), Madrid, Spain; 19 Research Group On Demography and Health, National Faculty of Public Health, University of Antioquia, Medellín, Colombia; 20 Cancer Risk Factors and Life-Style Epidemiology Unit, Institute for Cancer Research, Prevention and Clinical Network - ISPRO, Florence, Italy; 21 Epidemiology and Prevention Unit, Fondazione IRCCS Istituto Nazionale Dei Tumori Di Milano, Milan, Italy; 22 Department of Clinical and Biological Sciences, University of Turin, Turin, Italy; 23 Department of Public Health and Clinical Medicine, Family Medicine, Umeå University, Umeå, Sweden; 24 Department of Epidemiology and Biostatistics, School of Public Health, Imperial College London, London, UK; 25 Department of Hygiene and Epidemiology, University of Ioannina School of Medicine, Ioannina, Greece; 26 International Agency for Research on Cancer – IARC/WHO, Lyon, France

**Keywords:** Body mass index, Breast cancer, Colorectal cancer, Cardiovascular disease, Diabetes, Comorbidity, Cumulative exposure, Survival, Cohort study

## Abstract

**Background:**

Body mass index (BMI) and cardiometabolic comorbidities such as cardiovascular disease and type 2 diabetes have been studied as negative prognostic factors in cancer survival, but possible dependencies in the mechanisms underlying these associations remain largely unexplored. We analysed these associations in colorectal and breast cancer patients.

**Methods:**

Based on repeated BMI assessments of cancer-free participants from four European countries in the European Prospective Investigation into Cancer and nutrition (EPIC) study, individual BMI-trajectories reflecting predicted mean BMI between ages 20 to 50 years were estimated using a growth curve model. Participants with incident colorectal or breast cancer after the age of 50 years were included in the survival analysis to study the prognostic effect of mean BMI and cardiometabolic diseases (CMD) prior to cancer. CMD were defined as one or more chronic conditions among stroke, myocardial infarction, and type 2 diabetes. Hazard ratios (HRs) and confidence intervals (CIs) of mean BMI and CMD were derived using multivariable-adjusted Cox proportional hazard regression for mean BMI and CMD separately and both exposures combined, in subgroups of localised and advanced disease.

**Results:**

In the total cohort of 159,045 participants, there were 1,045 and 1,620 eligible patients of colorectal and breast cancer. In colorectal cancer patients, a higher BMI (by 1 kg/m2) was associated with a 6% increase in risk of death (95% CI of HR: 1.02–1.10). The HR for CMD was 1.25 (95% CI: 0.97–1.61). The associations for both exposures were stronger in patients with localised colorectal cancer. In breast cancer patients, a higher BMI was associated with a 4% increase in risk of death (95% CI: 1.00–1.08). CMDs were associated with a 46% increase in risk of death (95% CI: 1.01–2.09). The estimates and CIs for BMI remained similar after adjustment for CMD and vice versa.

**Conclusions:**

Our results suggest that cumulative exposure to higher BMI during early to mid-adulthood was associated with poorer survival in patients with breast and colorectal cancer, independent of CMD prior to cancer diagnosis. The association between a CMD diagnosis prior to cancer and survival in patients with breast and colorectal cancer was independent of BMI.

**Supplementary Information:**

The online version contains supplementary material available at 10.1186/s12885-022-09589-y.

## Background

High body mass index (BMI), indicating overweight or obesity, is one of the five leading risk factors of the Global Burden of Disease Study 2017 [1], and is estimated to cause 4.0 million deaths per year [2]. While its prevalence continues to grow in most parts of the world, to date about 39% and 12% of the world population can be considered overweight (BMI ≥ 25 kg/m^2^) or obese (BMI ≥ 30 kg/m^2^), respectively [2, 3]. In the context of adverse BMI-related health outcomes, the link between excess body fatness and cancer development has been well-established and studies have also reported dose–response relationships between the years of life spent with high BMI and cancer risk [4, 5].

Overweight and obesity have furthermore been associated with overall and cancer mortality [2, 6, 7]. Among the most common obesity-related malignancies are cancers of the breast and colorectum. A meta-analysis of 82 cohort studies reported that the total and cancer-specific mortality in women with breast cancer was 41% and 35% higher in obese patients, respectively, and 7% and 11% higher in overweight patients compared to normal weight patients [8]. Another meta-analysis of five cohort studies confirmed this relationship in breast cancer patients and reported that time to death was shortened by 16% in overweight women when compared with women whose average BMI was less or equal to 22.5 kg/m^2^ [9]. Furthermore, mortality in colorectal cancer patients was increased by 25% for all causes of death and 22% for cancer-related deaths in obese compared to normal weight patients, according to a meta-analysis of 16 prospective studies [10].

However, the role of BMI in cancer survival and the underlying mechanisms are not yet sufficiently understood [11]. A literature review discussing obesity related carcinogenesis and cancer progression identified biological mechanisms including obesity-induced inflammation, oxidative stress and the metabolism of steroid hormones, insulin, insulin-like growth factor-1, leptin and adiponectin as potential drivers of this association [12]. Furthermore, comorbidities have been shown to play an important role in cancer survival. According to a meta-analysis of 13 studies, the overall mortality of colorectal cancer patients was 41% higher in patients with mild or moderate comorbidity and more than 2 times higher in patients with severe comorbidity compared to those without comorbidity [13]. Cohort studies in breast cancer patients also found that cancer patients with comorbidity had poorer survival than those without comorbidity, reporting hazard ratios ranging from 1.1 to 5.8 [14–16]. While the prevalence of comorbidities such as cardiovascular disease (CVD) and type 2 diabetes (T2D) are high among cancer patients, a recent European cohort found that higher BMI further increases the risk of cancer-cardiometabolic multimorbidity [17]. Several studies found an association between obesity and cancer survival after controlling for comorbidities [8, 10, 18]. However, the role of BMI-related cardiometabolic conditions such as CVD and T2D in cancer survival and their contribution to BMI-related cancer mortality is still poorly understood [6, 11, 19]. In order to assess the impact of BMI and cardiometabolic diseases (CMD) on cancer survival and to investigate the importance of these risk factors in the context of the ongoing obesity epidemic, it is crucial to understand whether BMI and CMD are independently associated with cancer survival or contribute mutually.

The objective of this study is therefore to quantify the effect of cumulative BMI and cardiometabolic diseases, including CVD and T2D, prior to cancer on survival among breast and colorectal cancer patients, and to investigate the dependencies between these risk factors. We use cumulative BMI to minimize bias due to reverse causality, where weight loss due to cancer may have affected BMI prior to cancer diagnosis.

## Methods

### Study population and design

**Fig. 1:**
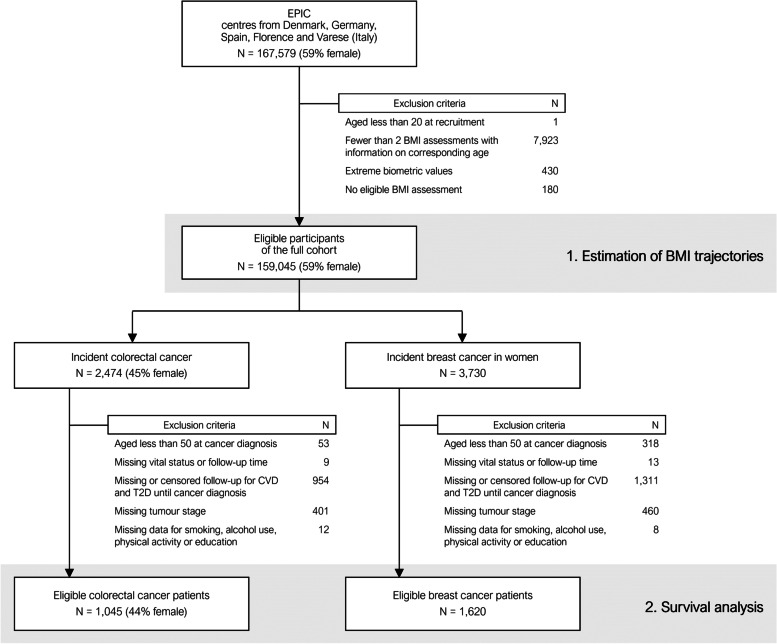
Flow chart with number of excluded and eligible participants of the study population

The European Prospective Investigation into Cancer and Nutrition (EPIC) is a cohort study of 519,978 volunteers from 23 centres in 10 countries, who were recruited between 1992 and 2000. The study design and methods of the EPIC study have been described in detail elsewhere [20]. Our current analysis uses data of all EPIC centres from Denmark, Germany and Spain and two centres from Italy (Florence and Varese). These cohorts included mainly volunteers from the general population aged 35 to 65 years. Exceptions are Spain, where participants were mostly blood donors, and Denmark, where the age range of participants at enrolment was 50 to 65 years [20]. The design of our current analysis consists of two steps: First, we used data of the full cohort population to estimate individual BMI-trajectories across age. These trajectories were subsequently used to derive the predicted mean BMI between ages 20 to 50 years, which served as a measure of cumulative BMI during early to mid-adulthood for each participant. Second, we restricted the data to patients who went on to develop colorectal or breast cancer during follow-up and performed a survival analysis to estimate the effect of cumulative BMI and cardiometabolic comorbidities on cancer survival (Fig. 1).

### Data collection

Each EPIC centre collected questionnaire data on lifestyle and health factors and anthropometric measurements at enrolment [20]. Up to three weight assessments were available for each participant, including weight measurements at enrolment, self-reported weight at follow-up, which was obtained on average 5 years after enrolment [21], and self-reported weight at age 20, which was assessed retrospectively in the baseline questionnaire. The respective BMI was calculated using height that was measured at enrolment.

In this analysis, we defined CMD as the combination of one or more comorbidities among self-reported history of T2D and CVDs at recruitment into the EPIC cohort, and incident events of T2D and non-fatal CVDs during follow-up between 1992 and 2007. Incident cases of T2D were ascertained and verified at each participating centre by a combination of self-report, linkage to primary-care registers, secondary-care registers, medication use (drug registers), hospital admissions and mortality data, and national diabetes and pharmaceutical registries [22]. Incident cardiovascular events included the following diagnoses according to the International Classification of Diseases (ICD-10): Myocardial infarction (I21, I22), angina (I20) or other coronary heart disease (I23-I25), haemorrhagic stroke (I60-I61), ischaemic stroke (I63), unclassified stroke (I64) and other acute cerebrovascular events (I62, I65-69, F01) [23]. First non-fatal coronary events were ascertained by different methods depending on the follow-up procedures by centre, using active follow-up through questionnaires or linkage with morbidity and hospital registries, or both. Validation was performed by retrieving and assessing medical records or hospital notes, contact with medical professionals, retrieving and assessing death certificates, or verbal autopsy [23].

The EPIC cohort was followed up for cancer diagnoses using linkages with population-based cancer registries in Denmark, Italy, and Spain, and based on active follow-up in Germany. Patients were identified according to the International Classification of Diseases for Oncology (ICD-O-3) with the codes C50 for breast cancer and C18-C20 for colorectal cancer sites. Stage of disease at diagnosis as available from the different study centres was harmonized into categories for localised or advanced (regional and distant) tumours.

All-cause mortality was collected by study centres using record linkages with cancer registries, boards of health and death indices in Denmark, Italy and Spain or through active follow-up (inquiries by mail/telephone, municipal registries/regional health departments, physicians/hospitals) in Germany. The data used in the present study includes follow-up of study participants from baseline (1992–2000) until December 2009 to December 2013 for countries with record linkage. For Germany, the end of follow-up was the last known contact with study participants (December 2009).

Information on smoking (never or ever), level of education (primary, secondary or tertiary) and average lifetime use of alcohol (g/day) was retrieved from a standardised dataset of EPIC lifestyle questionnaires at enrolment [20]. Alcohol consumption was substituted by a variable for alcohol use at recruitment (g/day) from the EPIC dietary questionnaire for 13 cancer patients where data on lifetime use was missing. A variable for occupational and recreational physical activity was created by collapsing the summary index of physical activity derived from the questions used in EPIC into two categories (inactive or active) [24].

### Statistical analysis

#### Estimation of individual BMI-trajectories

In the first step of the analysis, we estimated individual-specific BMI-trajectories based on the repeated BMI assessments of each participant of the full cohort using a growth curve model. Participants aged younger than 20 years at recruitment, with fewer than two BMI assessments during follow-up, extreme anthropometric values [25], or no eligible BMI measurement after the exclusion of measurements taken in the year before a diagnosis of cancer were excluded (Fig. 1).

We used a nested linear mixed effects model with a quadratic polynomial of age, notated as

$$BM{I}_{ijk}={\beta }_{0}+{u}_{0k}+{v}_{0jk}+\left({\beta }_{1}+{u}_{1k}+{v}_{1jk}\right)\cdot Ag{e}_{ijk}+{\beta }_{2}\cdot Ag{e}_{ijk}^{2}+{\epsilon }_{ijk}$$

to model the BMI measurement *i* of a patient *j* from country *k* as a function of age where *u* and *v* denote the random intercept and slope. Separate models were fit for males and females. The resulting individual quadratic functions of age were used to derive the BMI-related variables of cumulative exposure before cancer diagnosis for each participant. The predicted mean BMI was defined as the integral of the BMI trajectory between ages 20 to 50 years divided by 30 years.

#### Survival analysis

In a second step, we only included participants with incident cancers of the colorectum or the female breast in the survival analysis. Further inclusion criteria were cancer diagnosis at age 50 or older, non-missing information on vital status and (non-zero) follow-up time, CVD and T2D follow-up until cancer diagnosis and availability of information on stage at diagnosis and other adjusting variables (Fig. 1).

Cox proportional hazard regression was used to estimate hazard ratios (HRs) and 95% confidence intervals (CIs) for mortality in breast and colorectal cancer patients with years since diagnosis as the time scale.

Cox analyses were performed for mean BMI and CMD separately and for both exposures combined. This allowed for a qualitative evaluation of dependencies between the two variables. The models were stratified by age at diagnosis of cancer (categories for 50–69 years and 70 years or older), country and sex (for colorectal cancer). Models were adjusted for smoking status, physical activity, alcohol consumption and educational level at recruitment. Subgroup analyses by stage at diagnosis were performed to investigate differential effects comparing patients with localised or advanced disease. The proportional hazards assumption was assessed using the Grambsch-Therneau test. Likelihood ratio tests were used to test if the model fit could be improved by including CMD in addition to BMI or, vice versa, by including BMI in addition to CMD. To analyse potential non-linear effects of mean BMI, we repeated the Cox analyses using the same adjustment factors (including CMD) and estimated penalised B-splines with four degrees of freedom for the BMI variable. The resulting models were compared to models with a constant effect of mean BMI using likelihood ratio tests. To explore potential biases that would be introduced depending on the mechanism of missing data, we compared patients with missing information on stage at diagnosis with patients with a diagnosis of localised and advanced stage disease regarding their patient characteristics and their survival based on Kaplan–Meier curves.

Statistical tests with *P*-values below or equal to 0.05 were considered statistically significant. All analyses were carried out using the R statistical software version 3.6.1. In particular, the package nlme version 3.1–140 for linear mixed models and the package survival version 3.1–12 for Cox proportional regression including the function pspline for penalised B-splines [26, 27].

## Results

Numbers of individuals at each stage of the study are shown in Fig. 1. A total of 159,045 participants contributed an average of 2.4 BMI assessments to the estimation of the BMI-trajectories. The predicted mean BMI between age 20 and 50 in the total cohort had an IQR of 22.8–26.7 kg/m2 with a median of 23.9 kg/m^2^ in women and 25.4 kg/m^2^ in men.

The study included 1,620 breast cancer patients and 1,045 colorectal cancer patients. Over a median follow-up time of 9.48 years (IQR 5.42–12.50), 377 breast cancer patients and 509 colorectal cancer patients died. Cancer-site specific characteristics and mortality of the study population are reported in Table 1. Distributions of BMI, CMD and other patient characteristics were largely consistent across tumour stages and are presented in Table S 1. The Kaplan–Meier estimator of survival of colorectal and breast cancer patients by stage of disease of diagnosis is shown in Figure S 1.

**Table 1:** Patient characteristics in colorectal and breast cancer cases

	Colorectal cancer				Breast cancer			
	Total population		Deaths		Total population		Deaths	
	n	%	n	%	n	%	n	%
Total	1045		509	48.7	1620		377	23.3
Person years [person years]	7571.6		1983.4		16,349.2		2779.7	
Follow-up duration [years]^a^	6.93 (2.83, 11.20)				10.38 (7.04, 13.14)			
Female	461	44.1	211	45.8	1620	100.0	377	23.3
Male	584	55.9	298	51.0				
Age at cancer diagnosis [years]^a^	63.4 (59.0, 67.3)				60.6 (56.4, 64.8)			
50–69	921	88.1	448	48.6	1523	94.0	350	23.0
> 70	124	11.9	61	49.2	97	6.0	27	27.8
Tumour stage at diagnosis								
Localised	532	50.9	156	29.3	1032	63.7	152	14.7
Advanced	513	49.1	353	68.8	588	36.3	225	38.3
Predicted mean BMI [kg/m^2^]^a^	25.3 (23.5, 27.4)				23.6 (22.1, 25.5)			
< 25	480	45.9	232	48.3	1115	68.8	255	22.9
25–29.9	491	47.0	231	47.0	430	26.5	105	24.4
> = 30	74	7.1	46	62.2	75	4.6	17	22.7
Cardiometabolic disease	148	14.2	77	52.0	128	7.9	34	26.6
Myocardial infarction or stroke	57	5.5	29	50.9	39	2.4	15	38.5
Type 2 diabetes	104	10.0	54	51.9	92	5.7	21	22.8
Smoking								
Never	396	37.9	189	47.7	936	57.8	180	19.2
Ever	649	62.1	320	49.3	684	42.2	197	28.8
Physical activity								
Active	808	77.3	388	48.0	1249	77.1	291	23.3
Inactive	237	22.7	121	51.1	371	22.9	86	23.2
Alcohol consumption [g/day]^a^	11.9 (3.7, 29.5)				5.3 (1.5, 11.3)			
Education								
Primary	498	47.7	240	48.2	747	46.1	165	22.1
Secondary	353	33.8	176	49.9	659	40.7	165	25.0
Tertiary	194	18.6	93	47.9	214	13.2	47	22.0
Country								
Denmark	444	42.5	262	59.0	735	45.4	234	31.8
Germany	246	23.5	91	37.0	331	20.4	48	14.5
Italy	70	6.7	26	37.1	250	15.4	41	16.4
Spain	285	27.3	130	45.6	304	18.8	54	17.8

^a^continuous variables reported as median (interquartile range)

### Colorectal cancer

**Fig. 2:**
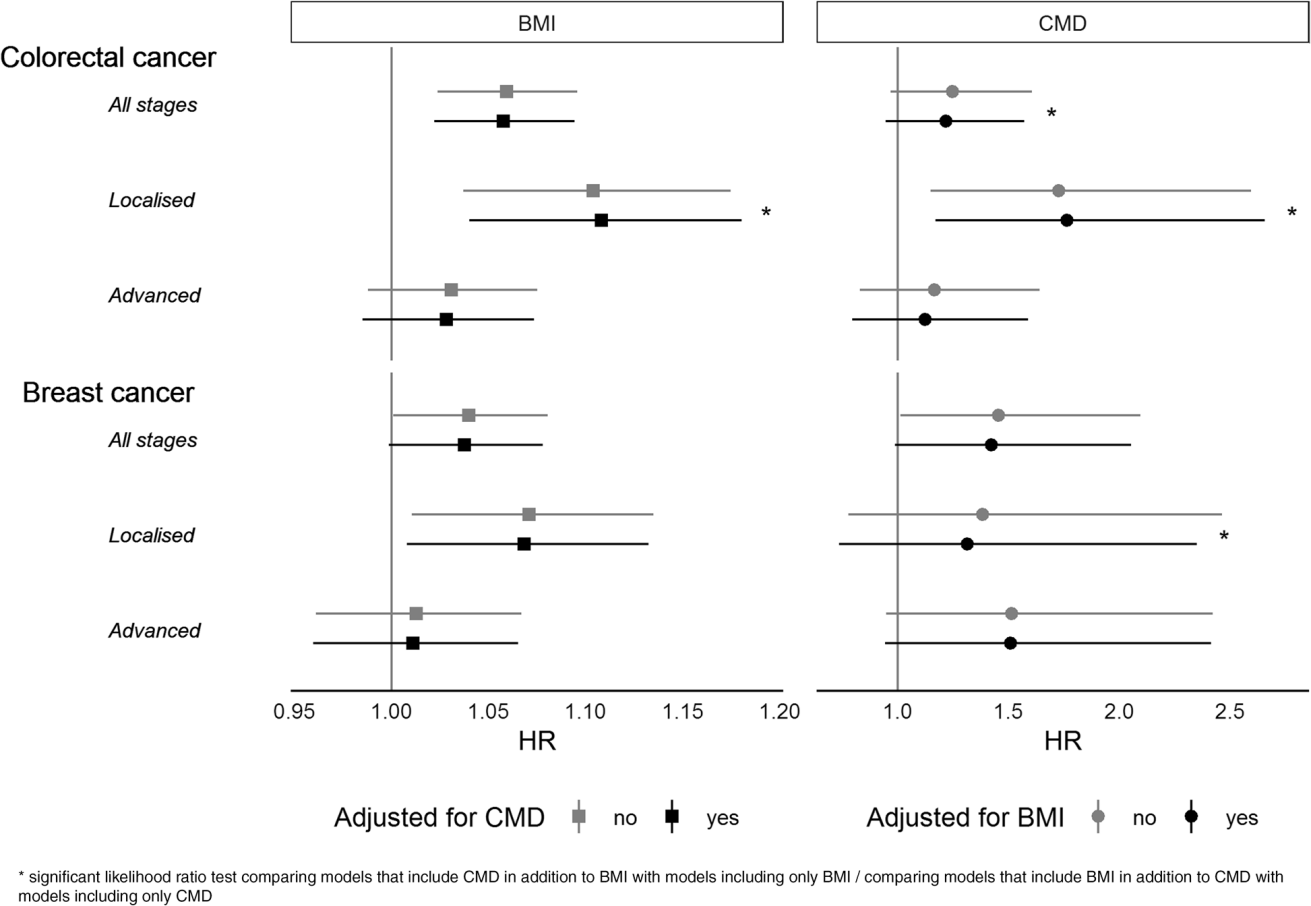
Hazard ratios of mean BMI and CMD for mortality from adjusted Cox proportional regression

According to multivariable adjusted survival analysis, a higher mean BMI (in incremental steps of 1 kg/m2) was associated with a 6% increase in risk of death in colorectal cancer patients (Table 2, Fig. 2). Results somewhat differed by stage of disease at diagnosis. Higher mean BMI was associated with a 10–11% higher mortality in localised colorectal cancers (95% CI of HR 1.04–1.17). The association between BMI and survival with advanced stage disease was less pronounced and included the null (HR 1.03, 95% CI 0.99–1.08). The estimates and confidence intervals of the effect of BMI remained similar after additional adjustment for CMD.

**Table 2:** Adjusted hazard ratios and 95% confidence intervals of BMI and CMD for mortality in breast and colorectal cancer patients by stage at diagnosis

		BMI (continuous, per 1 kg/m^2^)			CMD (reference: no CMD)		
	n	Adjusted for CMD	HR (95%-CI)	*P*-value (LR test)	Adjusted for BMI	HR (95%-CI)	*p*-value (LR test)
Colorectal cancer							
All stages	1045	No	1.06 (1.02–1.10)*		No	1.25 (0.97–1.61)	
		Yes	1.06 (1.02–1.09)*	0.065	Yes	1.22 (0.94–1.57)	0.002*
Localised	532	No	1.10 (1.04–1.17)*		No	1.73 (1.15–2.59)*	
		Yes	1.11 (1.04–1.18)*	0.031*	Yes	1.76 (1.17–2.66)*	0.002*
Advanced	513	No	1.03 (0.99–1.08)		No	1.17 (0.83–1.64)	
		Yes	1.03 (0.98–1.07)	0.683	Yes	1.12 (0.80–1.59)	0.213
Breast cancer							
All stages	1620	No	1.04 (1.00–1.08)*		No	1.46 (1.01–2.09)*	
		Yes	1.04 (1.00–1.08)*	0.069	Yes	1.42 (0.99–2.05)	0.135
Localised	1032	No	1.07 (1.01–1.13)*		No	1.38 (0.78–2.46)	
		Yes	1.07 (1.01–1.13)*	0.371	Yes	1.31 (0.74–2.35)	0.009*
Advanced	588	No	1.01 (0.96–1.07)		No	1.51 (0.95–2.42)	
		Yes	1.01 (0.96–1.07)	0.103	Yes	1.51 (0.94–2.41)	0.513

Cox proportional regression stratified by sex (for colorectal cancer), age and country and adjusted for smoking, physical activity, alcohol consumption and education

BMI: Predicted mean body mass index between age 20 and 50, continuous

CMD: Diagnosis of cardiometabolic diseases including myocardial infraction, stroke or type 2 diabetes

*P*-values of likelihood ratio tests (LR test) comparing models that include CMD in addition to BMI with models including only BMI / comparing models that include BMI in addition to CMD with models including only CMD

^*^Statistically significant on a significance-level of *p* <  = 0.05

The HR of CMD compared to no CMD was 1.25 for survival among colorectal cancer patients of all stages, but with a confidence interval including the null. Among patients with localised colorectal cancer, CMD was associated with a 73% (95% CI of HR 1.15–2.59) higher mortality. Adjustment for BMI lead to a slight increase of the effect estimate (HR 1.76, 95% CI 1.17–2.66). The HR of CMD compared to no CMD for survival in colorectal cancer patients with advanced stage was 1.17 (95% CI 0.83–1.64) without adjustment for BMI and 1.12 (95% CI 0.80–1.59) in the adjusted model.

Likelihood ratio tests suggested that adding BMI as a covariate to the model that included CMD improved model fit for colorectal cancer in the total sample (*p* = 0.002) and for localised stage (*p* = 0.002). Adding CMD to the model with BMI improved model fit for localised colorectal cancer (*p* = 0.03).

### Breast cancer

In breast cancer patients, a higher mean BMI (in incremental steps of 1 kg/m2) was associated with a 4% increase in risk of death (Table 2, Fig. 2). As for colorectal cancer, subgroup analyses by stage of disease at diagnosis showed that the association between BMI and mortality was stronger in patients with localised breast cancer. Higher mean BMI was associated with a 7% (95% CI of HR 1.01–1.13) higher mortality for localised breast cancers, while associations among patients with advanced stage disease were close to the null. The estimates and confidence intervals of the effect of BMI remained similar after additional adjustment for CMD.

The presence of CMD increased the risk of death in the total sample of breast cancer patients (HR 1.46, 95% CI 1.01–2.09). Estimates were fairly similar in sub-group analysis by stage, but confidence intervals included the null with/without additional adjustment for BMI.

Likelihood ratio tests suggest that adding BMI as a covariate to the model that included CMD improved model fit for localised breast cancer (*p* = 0.009).

The global Grambsch-Therneau tests showed no violation of the non-proportionality assumption for any of the multivariate Cox models.

The analysis of non-linear effects of mean BMI modelled with penalised B-splines did not show significant improvements of the model fit compared to the continuous effect of mean BMI (Figure S 2).

A description of patient characteristics by stage at diagnosis comparing patients with available information on tumour stage and patients with missing tumour stage is shown in Table S 2. Most patients with missing information on stage at diagnosis were from the Danish study centres. The median age was 4 years older and the prevalence of T2D and smoking was higher compared to patients with a diagnosis of either localised or advanced stage disease. The distribution of tumour staging information as available from the different study centres is shown in Figure S 3. The Kaplan–Meier estimator of survival of patients with missing information on stage at diagnosis ran approximately midway between the curves for localised or advanced stage disease (Figure S 1).

## Discussion

This analysis of data from the EPIC cohort showed that cumulative exposure to higher levels of BMI during early to mid-adulthood (ages 20 to 50 years) was associated with poorer survival in patients with localised colorectal and breast cancer. This relationship was independent of a history of CMD at cancer diagnosis. In turn, a history of CMD at cancer diagnosis was associated with poorer survival in patients with localised colorectal cancer, again independent of BMI. Estimates of the association between CMD and breast cancer survival indicated a positive association for all tumour stages, but confidence intervals included the null, particularly in our stage specific analysis.

Our results agree with evidence from meta-analyses on the association between BMI and colorectal and breast cancer prognosis [8–10]. However, most studies that investigated associations between BMI and cancer survival compared mortality of overweight or obese patients with normal weight patients, assuming constant risks within BMI categories, and used a one-point in time BMI assessment at cancer diagnosis. Moreover, previous studies did not specifically investigate the dependencies between BMI and CMD on survival for these cancers.

A previous study of colorectal cancer survival in Norway did not find an association with higher pre-diagnostic BMI per 5 kg/m^2^ increase [28]. However, a recent Swedish study investigating the effect of overweight during adulthood reported a 37% increased mortality in colorectal cancer patients and a 29% increased mortality in breast cancer patients with higher BMI per 2.65 kg/m^2^ increase [29]. A recent meta-analysis reported a significant dose–response relationship between the mean BMI during early and mid-adulthood and death from breast cancer, with a pooled hazard ratio of 1.31 (95% CI: 1.07–1.60), but no such association in colorectal cancer patients [9]. The results of our study reflect these findings, implying that associations between BMI and cancer survival can be observed for higher BMI per unit-wise increases and may not be limited to obese patients.

Our study adds further insights into the mechanisms that link higher cumulative BMI to mortality in breast and colorectal and breast cancer patients and suggest that increased risk of dying in patients with localised stage disease cannot be explained by comorbidities. We considered pre-diagnostic cases of T2D, myocardial infarction and stroke, representing underlying medical conditions that might be related to exposure to high levels of BMI and lead to worse survival. The estimated association of BMI with survival did not change after adjustment for CMD and was therefore independent of these risk factors in our analyses of colorectal and breast cancer patients. This qualitative assessment of the role of CMD in BMI-associated cancer survival strengthens the evidence of a possible direct impact of BMI that is not caused by a mediating effect of other BMI-related outcomes. A causal mediation analysis, which we performed on the subsample of colorectal cancer patients with localised stage disease, suggested that the total effect of cumulative BMI on survival was not mediated by cardiometabolic comorbidities prior to cancer.

While we observed suggestive associations between CMD and cancer mortality, which has also been observed in previous research [14], the confidence intervals were wide and included the null for most of the subgroups. A previous study of breast cancer patients showed that the effects of comorbidities on survival varied according to treatment, showing that breast cancer specific mortality was not related to a history of diabetes or myocardial infarction among patients receiving radiation and chemotherapy in contrast to patients who did not receive these treatments [30]. The lack of treatment information in our study could therefore be an explanation for unprecise effect estimates for CMD as a prognostic factor. Furthermore, patients with pre-diagnostic CVD included in our study are patients that had a non-fatal CVD event and lived on to develop cancer. These patients are likely to be subject to increased medical monitoring and receiving treatment that could decrease their subsequent risk of a cardiovascular event.

Little evidence for associations between BMI and cancer survival were found in colorectal and breast cancer patients with advanced stage disease. This could be due to the generally lower survival rate of patients with advanced disease, which leads to a smaller relative effect measure for the additional influence of BMI on mortality. This has been observed before for BMI in colorectal cancer [31] and in breast cancer [32].

It is important to discuss the epidemiologically observed link between BMI and cancer mortality in relation to the time period of body weight measurement (often referred to as pre-, peri- and post-diagnostic body weight) [6, 19]. While BMI around time of diagnosis or after diagnosis can be used as a prognostic factor of cancer outcome [6], it may not be ideal to assess aetiological links between BMI and cancer progression [28, 33]. A review on the epidemiological findings regarding obesity and cancer mortality argues that information on lifetime history of BMI can improve the interpretation of effect estimates for obesity-related cancer survival [33].

This current prospective cohort study has several strengths, as it includes repeated BMI assessments, data on lifestyle and health factors and validated information on incident CVD and T2D, that were collected before cancer diagnosis. The longitudinal structure of our data provides a clear distinction between periods of exposure and risk. The use of cumulative BMI minimizes bias due to reverse causality, where weight loss due to cancer may have affected BMI prior to cancer diagnosis. Furthermore, the estimation of individual BMI-trajectories based on repeated BMI assessments allowed us to derive measures of pre-diagnostic BMI that reflect the same period of early adulthood for all cancer patients, reducing the risk of misclassification compared to a single BMI assessment. The large data set that was used for the estimation of the BMI trajectories, which includes participants of the full cohort before restriction to patients who went on to develop cancer during follow-up, allows for a robust estimation of the growth curve models.

The findings of this study should be interpreted in light of its limitations. Since we did not include information on cause of death in our study, we cannot know if the excess deaths in patients with higher levels of BMI are related to an accelerated cancer progression or due to other BMI-related complications. However, it cannot be assumed that records on causes of death would allow for such distinction. Furthermore, we were not able to adjust for cancer treatment, as this information is not collected in the EPIC cohort. Treatment information should be considered in future analyses to investigate if effects of BMI and comorbidities on cancer mortality are related to suboptimal cancer treatment or treatment success. Comorbidities have been reported to be associated with a lower chance of receiving and completing standard cancer treatments [14]. Potential improvements in health behaviours after diagnosis of incident CMD or cancer could not be accounted for. However, any such changes would most likely have led to an underestimation of observed risks. Patients with missing data in one of the covariates were excluded from the present analysis, with the largest proportion of missing data being tumour stage at diagnosis. Patient characteristics were not fully consistent between patients with available information on tumour stage and patients with missing tumour stage, which in part can be explained by heterogeneity of the sub-cohorts of the different EPIC centres with different percentages of missing data. This heterogeneity also reflected in a higher mortality among Danish patients. The missing data analysis of the Kaplan–Meier curves did not indicate that the missing values are informative. However, further sensitivity analyses could allow for a better understanding of this heterogeneity and how to adequately account for it in the survival analysis. Multiple imputation of missing data could be included in future analyses to obtain more precise effect estimates than what can be achieved in a complete case analysis. Only self-reported weight at follow-up and at age 20 was available. However, in the EPIC-Norfolk study (UK Cambridge center of EPIC) a high correlation between self-reported and measured weight data has been shown (*r* = 0·97 in men and *r* = 0·98 women) [34], suggests that ranking of participants according to self-reported weight was adequate.

## Conclusions

Cumulative exposure to higher levels of BMI during early to mid-adulthood (ages 20 to 50 years) was associated with poorer survival in patients with breast and colorectal cancer, especially for localised disease, independent of CMD prior to cancer diagnosis. A history of CMD at cancer diagnosis was in turn associated with poorer survival in patients with localised colorectal cancer, again independent of BMI. Associations between CMD and breast cancer survival were not significant in our stage specific analysis. These results suggest that BMI has a direct effect on cancer survival that is not mediated by pre-diagnostic CMD and may contribute to improved prognostic stratification in cancer patients affected by cardiometabolic comorbidities.

## Supplementary Information

**Additional file 1.**

## Data Availability

Access to the datasets analysed in our study requires formal approval by the EPIC principal investigators. Instructions for submitting an application for access to EPIC data and/or biospecimens are available at http://epic.iarc.fr/access/index.php.

## Abbreviations

BMI: Body Mass Index
CI: Confidence interval
CVD: Cardiovascular disease
CMD: Cardiometabolic diseases
EPIC: European Prospective Investigation into Cancer and nutrition
HR: Hazard ratio
IQR: Interquartile range
T2D: Type 2 diabetes
