# Supplementary material for: Impact of cumulative body mass index and cardiometabolic diseases on survival among patients with colorectal and breast cancer: a multi-centre cohort study
Source: BMC Cancer. 2022 May 14;22:546. doi: 10.1186/s12885-022-09589-y (PMC9107127; doi:10.1186/s12885-022-09589-y)
Supplement: Supplementary file 1 — Additional file 1. [file 12885_2022_9589_MOESM1_ESM.pdf]

Table S 1 Description of the study population of breast and colorectal cancer patients by stage at diagnosis

	Colorectal cancer			Breast cancer		
	All stages	Localised	Advanced	All stages	Localised	Advanced
Person years	7571.6	4735.1	2836.5	16349.2	10723.4	5625.9
Follow-up duration [years]*	6.93 (2.83, 11.20)	9.03 (5.67, 12.00)	3.89 (1.71, 9.63)	10.38 (7.04, 13.14)	10.54 (7.46, 13.38)	10.05 (6.38, 12.64)
Number of cases	1045	532	513	1620	1032	588
Deaths (%)	48.7	29.3	68.8	23.3	14.7	38.3
Female (%)	44.1	43.2	45.0	100.0	100.0	100.0
Male (%)	55.9	56.8	55.0			
Age at cancer diagnosis [years]*	63.4 (59.0, 67.3)	63.3 (58.9, 67.3)	63.5 (59.0, 67.5)	60.6 (56.4, 64.8)	60.6 (56.2, 64.7)	60.7 (56.6, 65.0)
Age at cancer diagnosis (%)						
50-69	88.1	87.6	88.7	94.0	94.4	93.4
70+	11.9	12.4	11.3	6.0	5.6	6.6
Pred. mean BMI [kg/m²]*	25.3 (23.5, 27.4)	25.5 (23.7, 27.4)	25.1 (23.3, 27.2)	23.6 (22.1, 25.5)	23.6 (22.1, 25.5)	23.6 (22.0, 25.5)
Pred. mean BMI (%)						
<25	45.9	44.0	48.0	68.8	68.8	68.9
25-29.9	47.0	49.4	44.4	26.5	26.5	26.7
>=30	7.1	6.6	7.6	4.6	4.7	4.4
Cardiometabolic disease (%)	14.2	15.2	13.1	7.9	8.2	7.3
Myocardial infarction or stroke	5.5	6.0	4.9	2.4	2.7	1.9
Type 2 diabetes	10.0	10.3	9.6	5.7	5.6	5.8
Smoking (%)						
Never	37.9	37.0	38.8	57.8	58.7	56.1
Ever	62.1	63.0	61.2	42.2	41.3	43.9
Alcohol consumption [g/day]*	11.9 (3.7, 29.5)	12.6 (4.1, 29.4)	11.3 (3.6, 29.5)	5.3 (1.5, 11.3)	5.0 (1.5, 10.9)	5.9 (1.8, 11.8)
Physical activity (%)						
Active	77.3	76.3	78.4	77.1	76.9	77.4
Inactive	22.7	23.7	21.6	22.9	23.1	22.6
Level of education (%)						
Primary	47.7	48.5	46.8	46.1	46.4	45.6
Secondary	33.8	32.7	34.9	40.7	39.4	42.9
Tertiary	18.6	18.8	18.3	13.2	14.1	11.6
Country (%)						
Denmark	42.5	36.8	48.3	45.4	39.2	56.1
Germany	23.5	25.9	21.1	20.4	24.7	12.9
Italy	6.7	6.6	6.8	15.4	15.5	15.3
Spain	27.3	30.6	23.8	18.8	20.5	15.6

* continuous variables reported as median (interquartile range)

Table S 2 Description of the dataset by stage at diagnosis before exclusion of patients with missing data

	Colorectal cancer			Breast cancer		
	Localised stage	Advanced stage	Missing stage	Localised stage	Advanced stage	Missing stage
Person years	4793.5	2862.5	2374.5	10776	5636.3	3608
Follow-up duration [years]*	9.03 (5.67, 12.00)	3.89 (1.71, 9.63)	6.96 (3.29, 7.96)	10.54 (7.46, 13.38)	10.05 (6.38, 12.64)	7.79 (6.68, 8.97)
Number of cases	538	519	401	1038	590	460
Deaths (%)	29.2	68.6	44.4	14.8	38.3	18.3
Female (%)	43.3	45.1	40.1	100.0	100.0	100.0
Male (%)	56.7	54.9	59.9			
Age at cancer diagnosis [years]*	63.4 (59.0, 67.3)	63.5 (59.0, 67.4)	67.2 (62.6, 71.3)	60.6 (56.2, 64.7)	60.7 (56.6, 65.0)	64.8 (60.9, 69.6)
Age at cancer diagnosis (%)						
50-69	87.2	88.6	68.3	94.2	93.4	77.4
70+	12.8	11.4	31.7	5.8	6.6	22.6
Predicted mean BMI [kg/m²]*	25.5 (23.7, 27.4)	25.2 (23.3, 27.3)	24.9 (23.2, 26.7)	23.6 (22.1, 25.5)	23.6 (22.0, 25.5)	23.3 (21.9, 25.4)
Predicted mean BMI (%)						
<25	43.9	47.4	51.4	68.6	68.8	71.1
25-29.9	49.6	44.5	42.6	26.6	26.8	25.4
>=30	6.5	8.1	6.0	4.8	4.4	3.5
Cardiometabolic disease (%)	15.2	13.1	27.2	8.5	7.3	16.5
Myocardial infarction or stroke	6.1	4.8	8.7	2.8	1.9	4.3
Type 2 diabetes	10.2	9.6	23.4	5.9	5.8	13.7
Smoking (%)						
Never	37.4	38.9	29.4	58.9	55.9	50.7
Ever	62.5	60.9	70.6	41.0	44.1	49.1
Missing (n)	1	1	0	1	0	1
Alcohol consumption [g/day]*	12.6 (4.1, 29.3)	11.6 (3.7, 30.0)	13.1 (5.2, 26.5)	5.0 (1.5, 11.0)	5.8 (1.8, 11.8)	6.2 (2.6, 12.3)
Physical activity (%)						
Active	76.6	78.4	77.8	76.7	77.3	87.6
Inactive	23.4	21.6	22.2	23.2	22.7	12.2
Missing (n)	0	0	0	1	0	1
Level of education (%)						
Primary	48.1	46.4	39.2	46.1	45.4	30.7
Secondary	32.3	34.5	40.4	39.4	42.7	57.4
Tertiary	18.6	18.1	20.4	14.1	11.5	12.0
Missing (n)	5	5	0	4	2	0
Country (%)						
Denmark	37.0	48.0	77.3	39.3	56.1	83.5
Germany	25.7	21.0	7.2	24.6	12.9	8.3
Italy	6.5	6.7	5.7	15.4	15.3	2.8
Spain	30.9	24.3	9.7	20.7	15.8	5.4

* continuous variables reported as median (interquartile range)

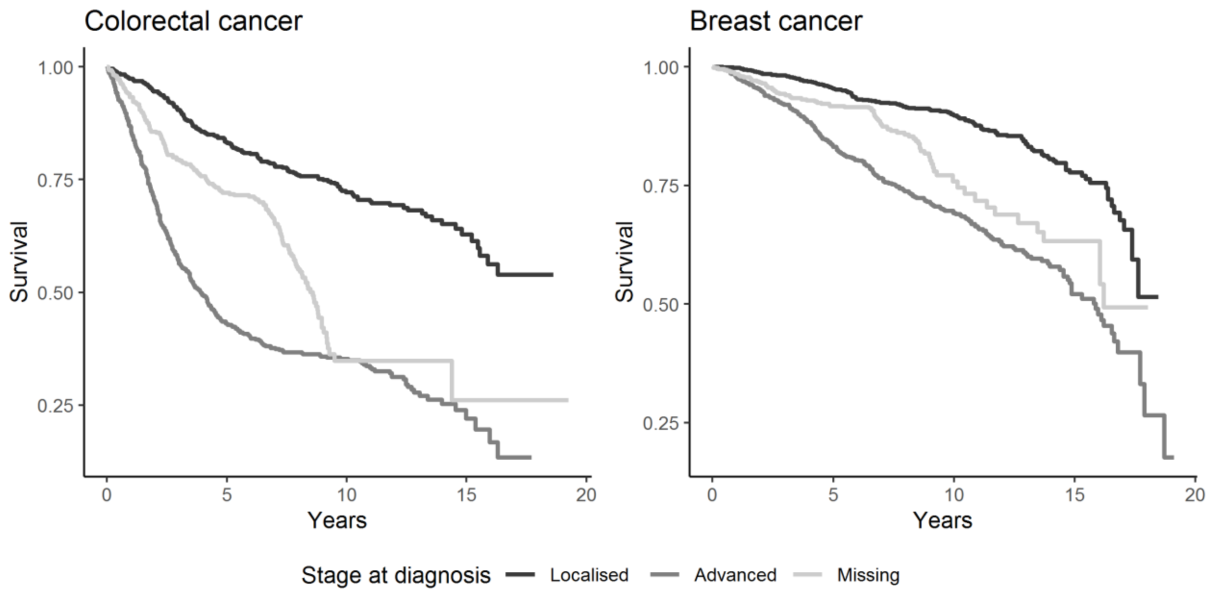

Figure S 1 Kaplan-Meier estimator of survival of colorectal and breast cancer patients by stage at diagnosis

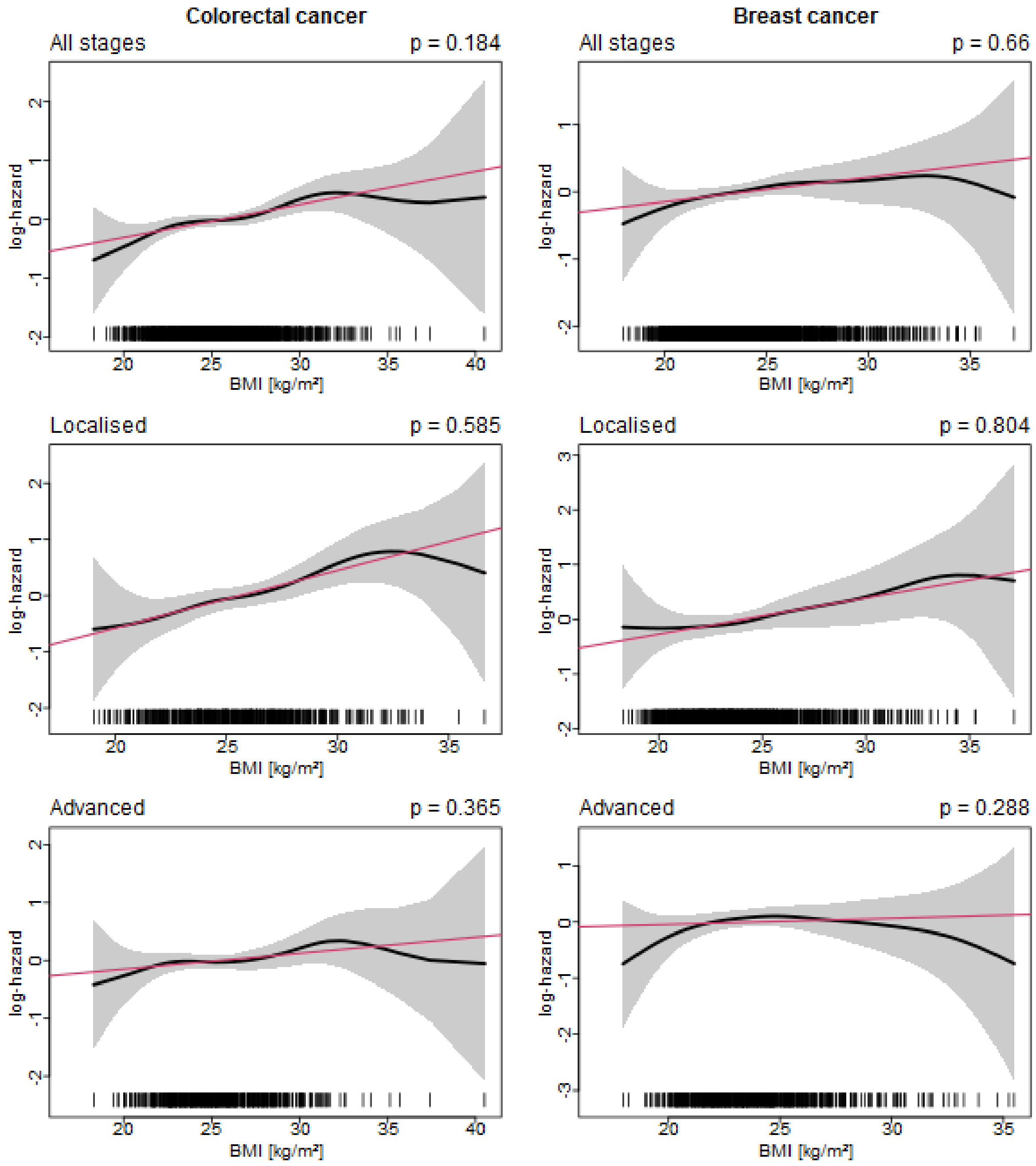

Figure S 2 Non-linear log-hazards of BMI (penalised B-spline with 4 degrees of freedom) and pointwise 95% confidence intervals (grey) compared to the estimated linear log-hazard per 1 unit increase in BMI (red) with p-values of the likelihood ratio test comparing the corresponding models. Cox proportional regression models of the effects of predicted mean BMI between ages 20 and 50 years on survival in breast and colorectal cancer patients were stratified by sex (for colorectal cancer), age and country and adjusted for cardiometabolic disease, smoking, physical activity, alcohol consumption and education.

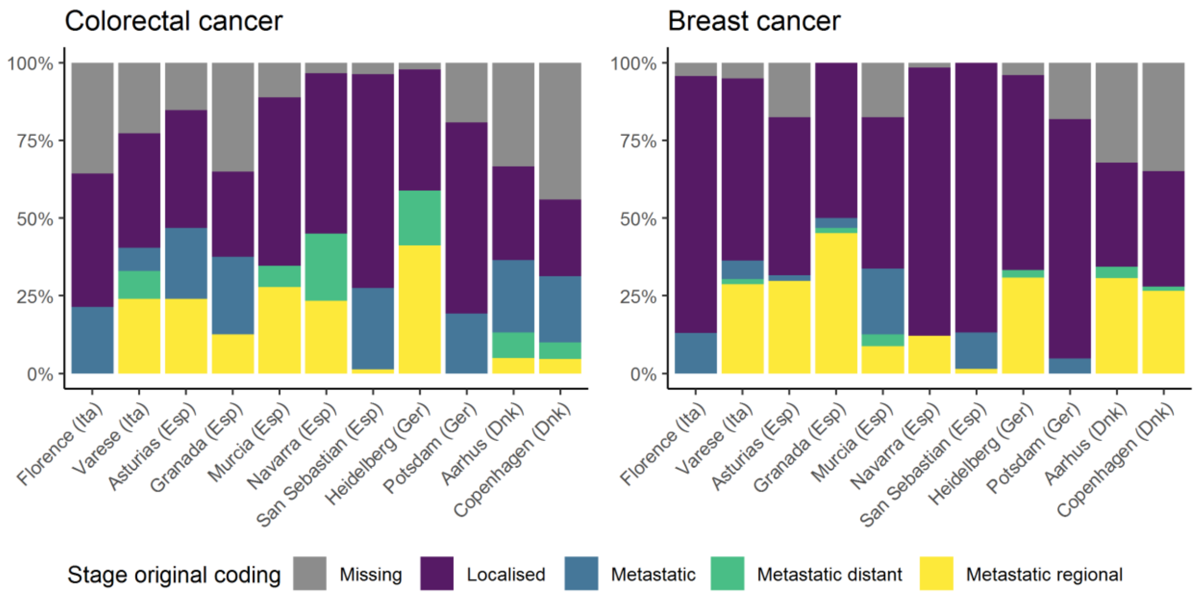

Figure S 3 Distribution of tumour staging information of breast and colorectal cancer patients by study centres from Italy (Ita), Spain (Esp), Germany (Ger) and Denmark (Dnk)
